# Identification and characterization of two distinct PPP1R2 isoforms in human spermatozoa

**DOI:** 10.1186/1471-2121-14-15

**Published:** 2013-03-18

**Authors:** Luis Korrodi-Gregório, Mónica Ferreira, Ana Paula Vintém, Wenjuan Wu, Thorsten Muller, Katrin Marcus, Srinivasan Vijayaraghavan, David L Brautigan, Odete A B da Cruz e Silva, Margarida Fardilha, Edgar F da Cruz e Silva

**Affiliations:** 1 Laboratory of Signal Transduction, Centre for Cell Biology, Biology Department, University of Aveiro, 3810-193, Aveiro, Portugal; 2 Functional Proteomics Department, Medizinisches Proteom-Center, Ruhr-University Bochum, Universitaetsstr. 150, 44809, Bochum, Germany; 3 Biological Sciences Biology Department, Kent State University, Kent, OH, 44242, USA; 4 Center for Cell Signaling, University of Virginia, School of Medicine, Charlottesville, VA, 22908, USA; 5 Laboratory of Neurosciences, Centre for Cell Biology, Biology Department; Health Sciences Department, University of Aveiro, 3810-193, Aveiro, Portugal; 6 Laboratory of Signal Transduction, Centre for Cell Biology, Biology Department; Health Sciences Department, University of Aveiro, 3810-193, Aveiro, Portugal; 7 Centro de Biologia Celular, Universidade de Aveiro, Aveiro, 3810-193, Portugal

**Keywords:** PP1, Phosphorylation, PP1 interacting protein, PPP1R2, PPP1R2P3, Pseudogene

## Abstract

**Background:**

Protein Ser/Thr Phosphatase PPP1CC2 is an alternatively spliced isoform of PPP1C that is highly enriched in testis and selectively expressed in sperm. Addition of the phosphatase inhibitor toxins okadaic acid or calyculin A to caput and caudal sperm triggers and stimulates motility, respectively. Thus, the endogenous mechanisms of phosphatase inhibition are fundamental for controlling sperm function and should be characterized. Preliminary results have shown a protein phosphatase inhibitor activity resembling PPP1R2 in bovine and primate spermatozoa.

**Results:**

Here we show conclusively, for the first time, that PPP1R2 is present in sperm. In addition, we have also identified a novel protein, PPP1R2P3. The latter was previously thought to be an intron-less pseudogene. We show that the protein corresponding to the pseudogene is expressed. It has PPP1 inhibitory potency similar to PPP1R2. The potential phosphosites in PPP1R2 are substituted by non-phosphorylable residues, T73P and S87R, in PPP1R2P3. We also confirm that PPP1R2/PPP1R2P3 are phosphorylated at Ser121 and Ser122, and report a novel phosphorylation site, Ser127. Subfractionation of sperm structures show that PPP1CC2, PPP1R2/PPP1R2P3 are located in the head and tail structures.

**Conclusions:**

The conclusive identification and localization of sperm PPP1R2 and PPP1R2P3 lays the basis for future studies on their roles in acrosome reaction, sperm motility and hyperactivation. An intriguing possibility is that a switch in PPP1CC2 inhibitory subunits could be the trigger for sperm motility in the epididymis and/or sperm hyperactivation in the female reproductive tract.

## Background

Spermatozoa are specialized cells that are highly compartmentalized, transcriptionally inactive and unable to synthesize new proteins. Protein phosphorylation is a post-translational mechanism that plays a crucial role in sperm physiology, controlling motility, capacitation, hyperactivated motility and the acrosome reaction [1,2]. Low sperm motility is one of the main causes of male infertility [2]. The biochemical mechanisms essential for the development of motility are still far from understood, however, serine/threonine protein phosphatase 1 (PPP1) and glycogen synthase kinase 3 (GSK3), have been recognized as components of the regulatory mechanism [3-5]. Three separate genes (α/A, β/δ/B and γ/C) encode the catalytic subunit of PPP1 (PPP1C). *PPP1CC* undergoes alternative splicing, giving rise to a ubiquitous isoform PPP1CC1 and a testis-enriched and sperm specific-isoform, PPP1CC2 [3,5]. PPP1CC2 is the only PPP1 isoform highly enriched in bovine, rhesus monkey and human sperm [3,5]. The phosphatase is distributed along the entire flagellum, including the mid-piece, consistent with a role in sperm motility, and also in the posterior and equatorial regions of the head, suggesting a role in the acrosome reaction [2,6,7]. The observation that PPP1C had a two-fold higher activity in immotile bovine caput epididymal sperm compared to mature motile caudal sperm is consistent with it being directly involved in sperm motility [3,5]. Moreover, inhibition of PPP1CC2 activity by the toxins okadaic acid or calyculin A induced and stimulated motility in caput and caudal sperm, respectively [3,5]. Homozygous knockout mice for *Ppp1cc* gene (a deletion of both isoforms) lead to sterility of male but not female mice. The sterility resulted from a combination of gross structural defects in spermatids that cause apoptosis and lack of spermiation [8,9]. The evolutionary conservation and the importance of Ser/Thr phosphatases in regulating flagellar motility, is highlighted by the involvement of a PPP1 homolog in the regulation of rooster sperm motility [10] and by the involvement of a Ser/Thr phosphatase in the regulation of microtubule sliding velocity in *Paramecium* and *Chlamydomonas*[11,12].

Regulation of PPP1 catalytic activity is mediated via binding to specific regulatory subunits, the PPP1 interacting proteins [13-15]. The extensive diversity of such interacting proteins and their tissue specificity makes them potential pharmacological targets [14]. To date, studies have shown that sperm do not appear to contain PPP1R1 (inhibitor-1, I1), whose activity is controlled by protein kinase A phosphorylation [3,5,16]. However, a PPP1R2-like activity seems to be present in sperm due to its sensitivity to GSK3 phosphorylation [3,5]. PPP1 and PPP1R2 are known to form a stable, catalytically inactive heterodimer, also known as MgATP-dependent phosphatase [17]. PPP1R2 can also bind and regulate PPP1C already complexed to other regulatory subunits [17]. Phosphorylation of PPP1R2 at Thr73 by GSK3 releases the inhibition and the phosphatase becomes active [18]. Immotile caput sperm contained six-fold higher GSK3 activity than motile caudal sperm [3,5]. The presence and activity of GSK3 in sperm has been further characterized, emphasizing its role in sperm motility regulation [19,20].

Recent experiments have demonstrated that sperm also contains the homologue of the yeast PPP1 binding protein, PPP1R7 (Sds22) and that the complex with PPP1CC2 is catalytically inactive [6]. Finally, a potent heat-stable inhibitor was identified as the orthologue of the mouse t complex testis-expressed gene Tctex5, also known as PPP1R11 (inhibitor 3, I3) [21]. The heat stability of these inhibitors is important for facilitating the purification, although no other function was found so far for this property. It was also shown that both inhibitors, PPP1R7 and PPP1R11, form a catalytically inactive multi-complex with PPP1CC2 in sperm [22].

Despite these findings whether sperm contain PPP1R2 remains undetermined. The purpose of this study was to determine if PPP1R2 was present in spermatozoa. We show here, for the first time, the presence and localization of PPP1R2 in human spermatozoa. Furthermore, we demonstrate the presence of PPP1R2P3, a new isoform of PPP1R2. The gene corresponding to PPP1R2P3 was previously designated as an intron-less pseudogene [23]. Our studies raise the intriguing possibility that the interplay between PPP1CC2/PPP1R2 and PPP1CC2/PPP1R2P3 could operate to regulate human sperm function.

## Results and discussion

### Identification of PPP1R2P3 - a novel PPP1R2 isoform in testis

The catalytic subunit isoforms PPP1CC1 and PPP1CC2 were used as baits to screen a human testis cDNA library by yeast two-hybrid [2,15]. From the *PPP1CC1* screening, 120 positive clones were recovered, one of which encoded the complete sequence of a novel isoform of the regulatory protein known as PPP1R2 or inhibitor 2 (I2). This novel clone is located on chromosome 5, aligns to the sequence ID:NR002168 and is classified as PPP1R2 pseudogene 3 (PPP1R2P3). While *PPP1R2* is present on chromosome 3 and is encoded by 5 exons, *PPP1R2P3* is an intronless gene and therefore was designated as a probable pseudogene by both NCBI and Ensembl databases. This clone has been deposited in the GenBank database under the ID:JF438008.1 [2,15]. The *PPP1R2P3* mRNA is also present in human testis cDNA library repository of the Mammalian Gene Collection (MGC) program (nucleotide ID: BC066922; protein ID: Q6NXS1) [24]. By searching Unigene (NCBI) for PPP1R2P3 specific expressed sequence tags (ESTs) 15 ESTs were obtained, of which 14 are from testis. These ESTs are specific for this pseudogene and cover close to 65% of the PPP1R2P3 nucleotide sequence including the CDS. Taken together, our results, the ESTs, and the MGC results [24], support the proposal that PPP1R2P3 is not a pseudogene and is indeed expressed in testis. Furthermore, by blast search we only found orthologs of this pseudogene in primate’s assembly genomes.

**Figure 1:**
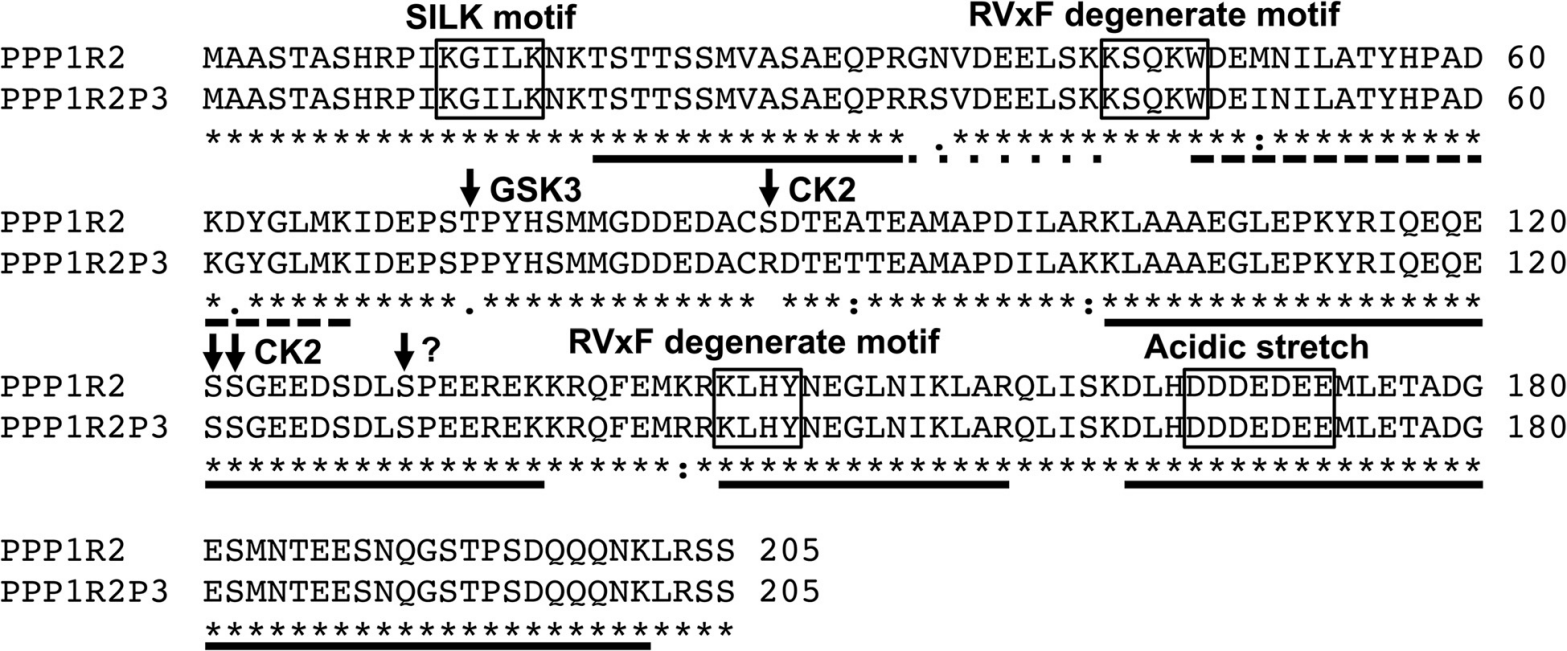
**ClustalW2 alignment of PPP1R2 and PPP1R2P3.** Protein sequences for PPP1R2 and PPP1R2P3 were obtained from Uniprot database. Sequences were submitted to a ClustalW2 alignment. Relevant motifs/regions for PPP1R2/PPP1R2P3 binding to PPP1C are shown in open boxes. Important phosphorylation sites are indicated with black arrows above the residues and with the respective known kinase. * represent high conservation, : and . represent low conservation in which the substituted residue has respectively more and less similar properties. Black horizontal bars below the protein sequences show the coverage of the peptides obtained by mass spectrometry. Black horizontal dash bar indicates the peptide that allowed for the distinction between PPP1R2P3 from PPP1R2. Black horizontal square dot bar indicates the peptide that allowed distinguishing PPP1R2.

*PPP1R2P3* has only 16 nucleotide substitutions (92.2%, identity) relative to PPP1R2, which correspond to 9 amino acid changes (95%, identity) in the translated sequence. Comparing the PPP1R2P3 protein sequence with that of the PPP1R2 using ClustalW2, we found that all the PPP1C binding regions are maintained (Figure 1). However, there are important differences between PPP1R2 and PPP1R2P3, in that the phosphorylation sites of the latter, for GSK3 (Thr73) and CK2 kinase (Ser87), appear as Pro and Arg, respectively (Figure 1).

### PPP1R2P3 interacts and inhibits PPP1C

**Figure 2:**
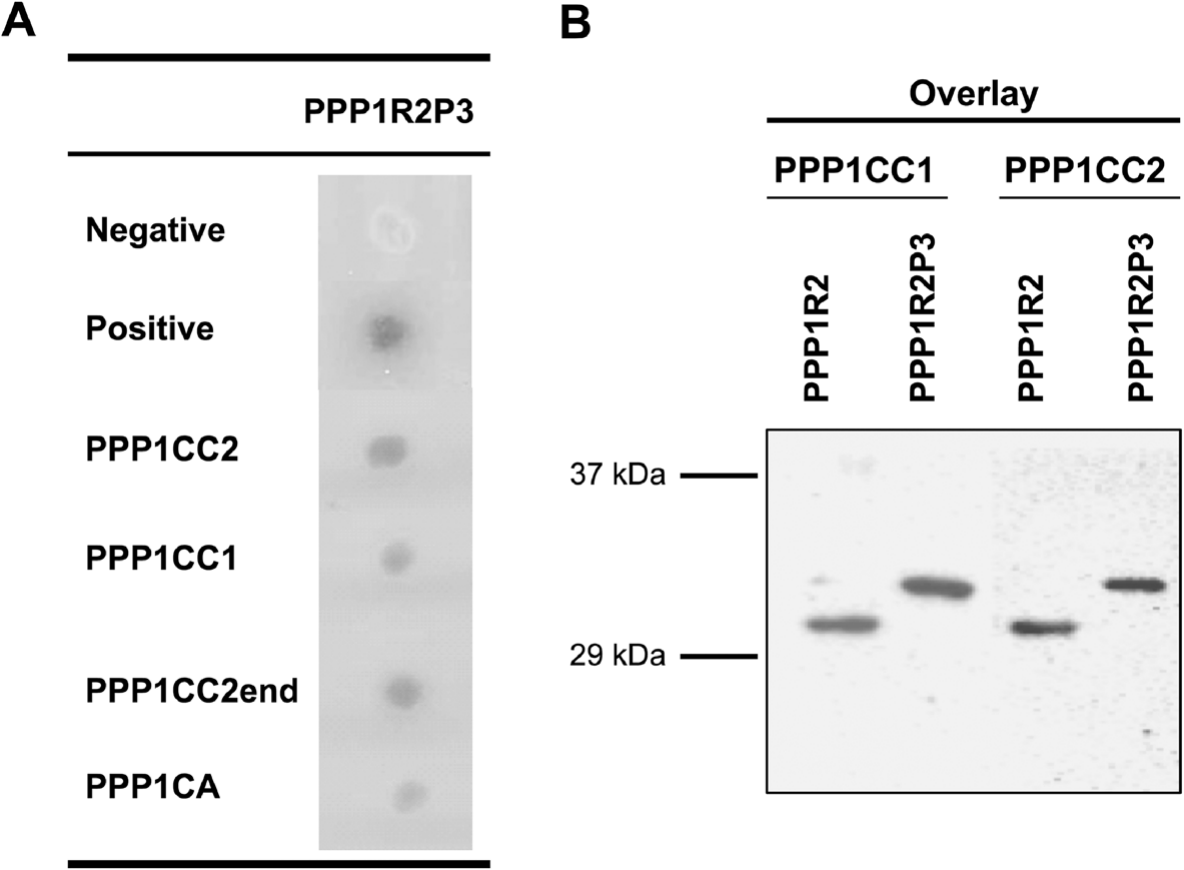
**Interaction of PPP1R2P3 with different PPP1 isoforms.** (**A**), Sequential transformation of yeast AH109 with bait plasmid (pAS2-PPP1CA, pAS2-PPP1CC1, pAS2-PPP1CC2, or pAS2-PPP1CC2end) and the prey plasmid pACT2-PPP1R2P3. pAS2-PPP1CC2end is the unique C-terminal tail of PPP1CC2 produced by alternative splicing of the PPP1CC gene. For negative and positive controls pAS2-1/pACT-2 and pVA3-1/pTD1-1 vectors were used, respectively (**B**), Overlay assay of PPP1R2 and PPP1R2P3. Commercial PPP1R2 and His-PPP1R2P3 were separated by SDS-PAGE, transferred to nitrocellulose and overlaid with recombinant PPP1CC1 or PPP1CC2, as indicated. Western blotting was performed with the respective specific antibodies.

To validate the yeast two-hybrid result we re-confirmed the PPP1R2P3 interaction with different PPP1C isoforms. The sequential yeast co-transformation, showed the interaction of PPP1R2P3 with PPP1CA, PPP1CC1 and PPP1CC2 and with the unique C-terminal of PPP1CC2 (PPP1CC2end) (Figure 2A). The apparent relative lack of specificity toward the PPP1C isoforms may arise given that in yeast co-transformation the interaction is forced and happens inside the nucleus. *In vivo*, the PPP1C isoforms have different tissue/cellular and subcellular localizations that would influence the PPP1R2P3 interaction. In order to compare the binding of PPP1R2 and PPP1R2P3 to PPP1CC isoforms, and demonstrate the interaction as direct, a blot overlay was performed (Figure 2B). A blot containing the same amount of commercial PPP1R2 or His-PPP1R2P3 was incubated with PPP1CC1 or PPP1CC2 and the interaction detected using the CBC3C and the CBC502 antibodies, respectively (Figure 2B). The binding of PPP1R2P3 to both PPP1CC isoforms was confirmed and the binding was similar to that of PPP1R2.

**Figure 3:**
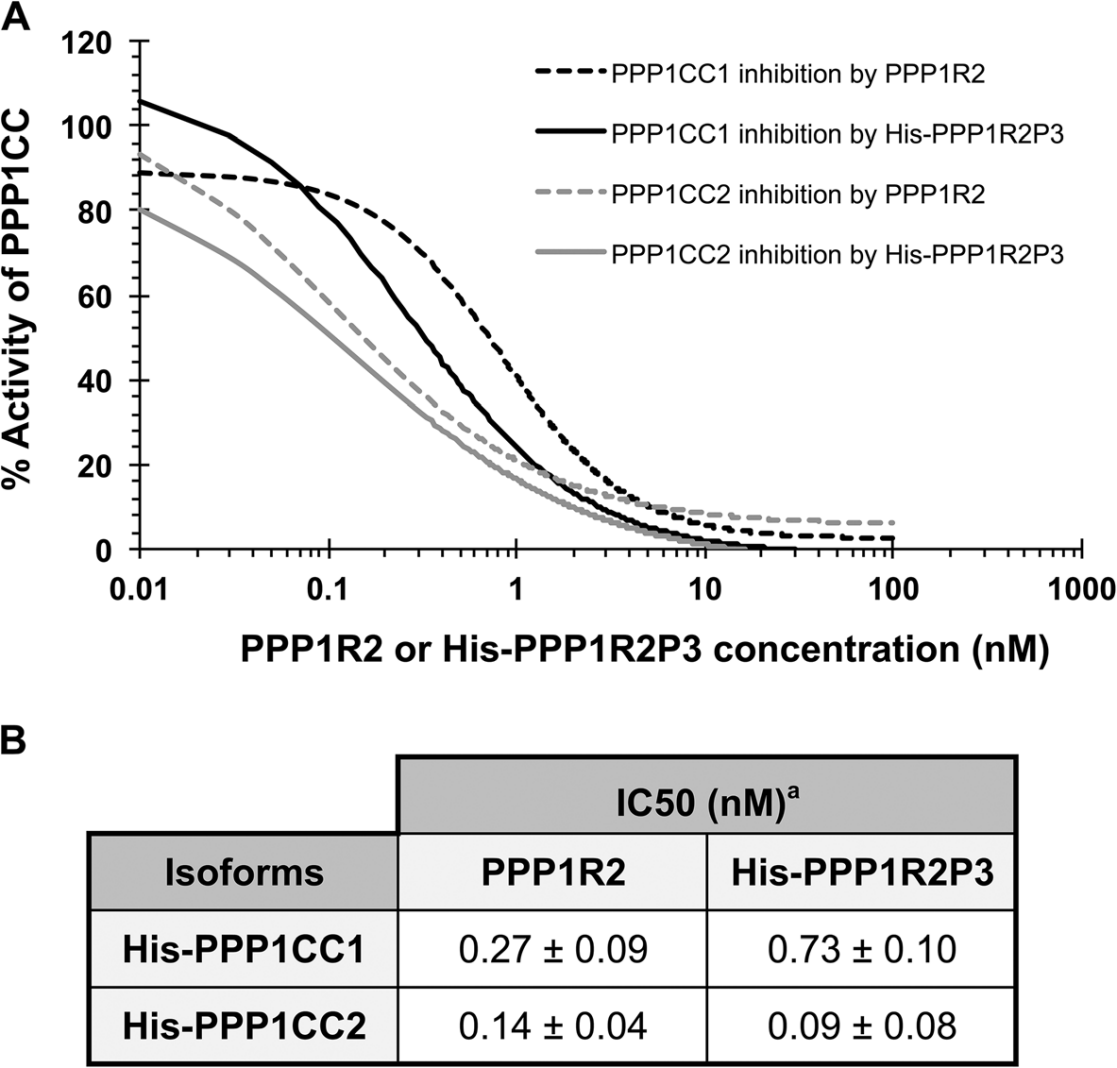
**PPP1R2 and PPP1R2P3 inhibit PPP1CC. (A)**, Graphical representation of PPP1CC1 and PPP1CC2 inhibition curves by commercial PPP1R2 and His-PPP1R2P3, using phosphorylase a as substrate. (**B**), Table showing the comparison of PPP1CC isoforms IC50s by PPP1R2 and PPP1R2P3 using the phosphorylase phosphatase assay. ^**a**^ The values are expressed as the mean ± S.E.M. of at least three independent experiments.

Since PPP1R2 is a potent heat-stable inhibitor of PPP1 in the nanomolar range [25], we decided to determine whether PPP1R2P3 is heat-stable, if it exhibited PPP1 inhibitory activity, and if so, what was the potency compared to that of PPP1R2. We determined the IC50 values of recombinant His-PPP1R2P3 for the PPP1CC isoforms using the standard phosphorylase phosphatase assay [26]. Results showed that PPP1R2P3 is a potent heat-stable inhibitor of PPP1C with a dose-response curve similar to the commercial PPP1R2 (Figure 3A) and IC50 values in the subnanomolar range (Figure 3B, PPP1CC1, 0.73 ± 0.10 nM and PPP1CC2, 0.09 ± 0.08 nM).

### GSK3 does not phosphorylate PPP1R2P3 *in vitro*

**Figure 4:**
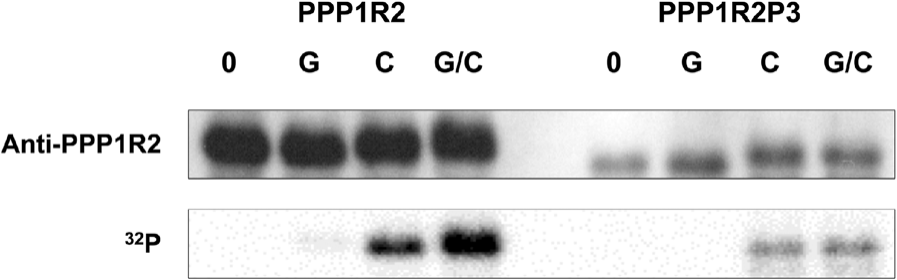
**Comparison of PPP1R2 and PPPR2P3 phosphorylation by GSK3 and CK2.** Recombinant PPP1R2 and PPP1R2P3 were incubated in kinase buffer with reactive ATP (0); or in the presence of GSK3β kinase (G), CK2 kinase (C) or both kinases (G/C), for 90 min at 30^o^C. Protein phosphorylation was detected by autoradiography (^32^P) following SDS-PAGE. Immunoreactivity detected with a specific sheep anti-PPP1R2 antibody is shown for comparative purposes.

Phosphorylation of PPP1R2 at Thr73 (in rabbit) by GSK3, relieves the inhibition of PPP1C and the complex PPP1C/PPP1R2 becomes active [18]. Also, phosphorylation by CK2 at the serines 87, 121 and 122 enhances Thr73 phosphorylation [27,28]. The absence of the GSK3 phosphorylation site, Thr73, and of the CK2 phosphorylation site, Ser87, in PPP1R2P3 led us to test the phosphorylation of PPP1R2P3 by these kinases. PPP1R2 and PPP1R2P3 were incubated in parallel reactions with GSK3, or CK2, or both, in the presence of 32P-ATP. PPP1R2 is known to be a poor substrate for GSK3 alone *in vitro*, but synergistic phosphorylation can be observed if phosphorylated by CK2 plus GSK3 [28]. In our hands this occurred with recombinant PPP1R2 (Figure 4, first panel). However, under the same conditions, no PPP1R2P3 phosphorylation by GSK3 was detected and the phosphorylation in the presence of both kinases was the same as with CK2 alone (Figure 4, second panel). The results indicate that there is no site for GSK3 phosphorylation in PPP1R2P3 and that CK2 is still able to phosphorylate but in other residue rather than Ser87.

### PPP1R2 and PPP1R2P3 are present in human ejaculated sperm

Based on previous biochemical studies it was suggested that sperm PPP1CC2 is regulated by a PPP1R2-like activity [3,5]. However, there was no definitive evidence for the presence of PPP1R2 in spermatozoa. Efforts to identify PPP1R2 at the protein level in testis and sperm have proven to be difficult because of the low amount of protein in soluble extracts and the quality of the available antibodies. PPP1R2 is an intrinsically unstructured protein (IUP) with a high proportion of charged and hydrophilic residues versus few hydrophobic residues [29]. Proteins such as PPP1R2 (predicted, 23 kDa), will migrate in SDS-PAGE at a position corresponding to a higher molecular mass than predicted by the primary sequence (i.e. 32 kDa) [30,31]. PPP1R2P3 has the same predicted molecular mass of PPP1R2 and in SDS-PAGE migrated at the same position of 32 kDa (Figure 2B). To deal with the low abundance and taking advantage of the fact that PPP1R2 is heat-stable, protein precipitation was used to concentrate the samples from heat-treated extracts [32]. So far only one report showed successful identification of PPP1R2 in heat-stable extracts of bull testis and mouse testis and sperm using a PPP1R2 antibody (rabbit anti-PPP1R2, see Methods) raised in rabbit against an affinity-purified peptide (^135^REKKRQFEMKRKLH^148^ from the mouse sequence) [33].

**Figure 5:**
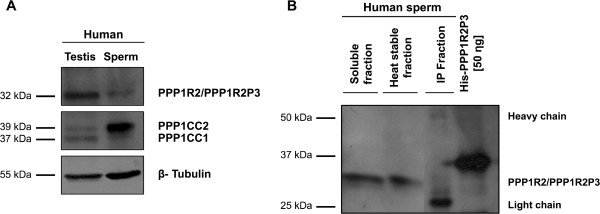
**PPP1R2/PPP1R2P3 are present in human testis and ejaculated sperm.** (**A**), A Western blot of human testis and sperm screening (50 μg) of PPP1R2/PPP1R2P3 and PPP1CC isoforms was performed using sheep anti-PPP1R2 and CBC3C antibodies. Extracts were prepared in 1%SDS. β-Tubulin was used as loading control. **(B**), PPP1R2/PPP1R2P3 was immunoprecipitated from a human sperm heat-stable extract with sheep anti-PPP1R2 antibody followed by Western blotting with the same antibody. Recombinant His-PPP1R2P3 (50ηg) and sperm lysate before boiling (soluble fraction) were used as controls. PPP1R2 was immunoprecipitated using dynabeads protein G. IP, immunoprecipitation.

We used a sheep polyclonal anti-PPP1R2 antibody, raised against purified rabbit PPP1R2 as immunogen to detect PPP1R2/PPP1R2P3 as a band around the expected size (32 kDa) in testis and sperm extracts (Figure 5A) [34]. To demonstrate the presence of PPP1CC in the same extracts, an antibody against the C-terminus of PPP1CC was used (CBC3C). We detected two bands in testis that correspond to PPP1CC1 and PPP1CC2, while only one band, PPP1CC2, was detected in sperm, as expected [3,5]. Moreover, since PPP1R2 and PPP1R2P3 are heat-stable inhibitors, we heated RIPA extracts by boiling. Western blotting revealed a band corresponding to PPP1R2/PPP1R2P3 at the expected size of 32 kDa (Figure 5B, second lane). To unequivocally demonstrate the existence of PPP1R2 and the new PPP1R2P3 isoform in human sperm we performed an immunoprecipitation of PPP1R2/PPP1R2P3 from five independent human ejaculated sperm samples (using anti-PPP1R2 antibodies (four using the sheep anti-PPP1R2 and one using the rabbit anti-PPP1R2) followed by mass spectrometry analysis. Immunoprecipitation was performed using heat-stable extracts of human ejaculates. We immunoprecipitated sufficient amounts of PPP1R2/PPP1R2P3 for detection of their peptides by mass spectrometry (Table 1). Using the Orbitrap Velos mass spectrometer we identified 41 unique MS/MS spectra corresponding to 11 different peptides of PPP1R2/PPP1R2P3. From these, 5 MS/MS spectra correspond to 2 peptides that match uniquely with PPP1R2, 3 MS/MS spectra correspond to a peptide that matches uniquely to PPP1R2P3 and the remaining correspond to common sequences (Table 1 and Figure 1). The overall sequence coverage was 64% for PPP1R2 and 57% for PPP1R2P3. The mascot scores for each protein were near 600 (additionally, spectra were manually evaluated). This is the first report of PPP1R2 and PPP1R2P3 recovery from multiple extracts of human sperm, using two different antibodies.

**Table 1:** Peptides identified by Orbitrap Velos mass spectrometry for PPP1R2, PPP1R2P3 or both and for PPP1CC2, after immunoprecipitation of human sperm samples with sheep or rabbit anti-PPP1R2 antibodies. aa, amino acids; pI, isoelectric point

**Protein name**	**Uniprot ID**	**MW (Da)**	**pI**	**Protein size (aa)**	**Coverage**	***Mascot score***
PPP1R2	IPP2_HUMAN	23.015	4.64	205	22.0%	74.12
***Peptide***		***Range (start-end)***	***Number of spectra***	***m/z meas.***	***z***	***Mascot score***
K.WDEMNILATYHPADKDYGLMK.I		47–67	3	848.39	3	47.40
K.TSTTSSMVASAEQPRGNVDEELSK.K		19–42	2	847.40	3	26.72
**Protein Name**	**Uniprot ID**	**MW (Da)**	**pI**	**Protein size (aa)**	**Coverage**	***Mascot score***
PPP1R2P3	IPP2M_HUMAN	23.048	4.82	205	7.3%	48.95
***Peptide***		***Range (start-end)***	***Number of spectra***	***m/z meas.***	***z***	***Mascot score***
K.WDEINILATYHPADK.G		47–61	3	595.96	3	48.95
**Protein Name**		**MW (Da)**	**pI**	**Protein size (aa)**	**Coverage**	***Mascot score***
Common to both PPP1R2 and PPP1R2P3					49.3%	546.34
***Peptide***		***Range (start-end)***	***Number of spectra***	***m/z meas.***	***z***	***Mascot score***
R.KLAAAEGLEPK.Y		103–113	7	563.83	2	70.38
R.IQEQESSGEEDSDLSPEER.E		116–134	5	1082.46	2	117.82
K.LAAAEGLEPK.Y		104–113	5	499.78	2	63.21
K.LHYNEGLNIK.L		146–155	4	400.88	3	53.17
R.KLHYNEGLNIK.L		145–155	4	664.87	2	51.25
K.LHYNEGLNIKLAR.Q		146–158	2	514.29	3	23.57
R.IQEQESSGEEDSDLSPEEREK.K		116–136	2	807.69	3	59.32
K.DLHDDDEDEEMLETADGESMNTEESNQGSTPSDQQQNK.L		164–201	1	1434.23	3	30.63
K.YRIQEQESSGEEDSDLSPEER.E		114–134	1	828.70	3	11.42
K.LAAAEGLEPKYR.I		104–115	1	439.91	3	15.97
K.TSTTSSMVASAEQPR.G		19–33	1	784.86	2	49.60
**Protein Name**	**Uniprot ID**	**MW (Da)**	**pI**	**Protein size (aa)**	**Coverage**	***Mascot score***
PPP1CC2	PP1G_HUMAN	38.518	5.78	337	15%	252.57
***Peptide***		***Range (start-end)***	***Number of spectra***	***m/z meas.***	***z***	***Mascot score***
R.VASGLNPSIQK.A		315–325	5	557.32	2	54.76
R.GVSFTFGAEVVAK.F		222–234	2	656.35	2	72.94
K.NVQLQENEIR.G		27–36	2	621.83	2	42.11
K.YPENFFLLR.G		114–122	1	599.82	2	39.6
K.LNIDSIIQR.L		7–15	1	536.31	2	43.16

Mass spectrometry data of the same immunoprecipitates showed that PPP1CC2 was also present. Five peptides out of 10 MS/MS spectra were identified, that matched PPP1CC2 with 15% coverage and a protein MASCOT score higher than 200 (manually evaluated). The results show that PPP1CC2 binds to PPP1R2/PPP1R2P3 in human spermatozoa (Table 1).

In extracts from ejaculated sperm, three independent peptides show post-translational modifications, identified as phosphorylation at Ser121, Ser122 and Ser127. However, they could not be assigned to either PPP1R2 or PPP1R2P3, given that both proteins are identical in that region. To our knowledge, this is the first time that phosphorylation of Ser127 has been reported for PPP1R2/PPP1R2P3. Tyrosine or threonine phosphorylations were not detected. The peptides obtained with phosphorylations correspond to only 7% of the total number of obtained peptides, strongly supporting the fact that PPP1R2/PPP1R2P3 are predominantly not phosphorylated.

### PPP1R2 subcellular-localization in human spermatozoa

**Figure 6:**
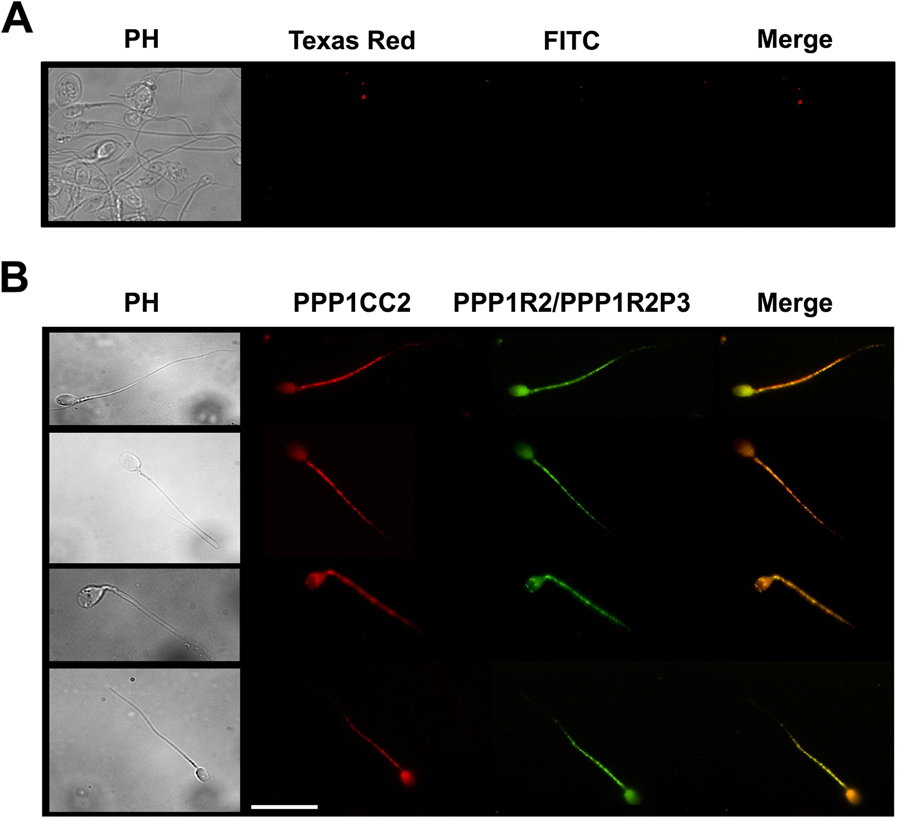
**Co-localization of PPP1CC2 and PPP1R2/PPP1R2P3 in morphologically normal and abnormal spermatozoa.** Human spermatozoa were labeled with rabbit anti-PPP1CC2 and sheep anti-PPP1R2 antibodies, and specific secondary antibodies conjugated with Texas-Red and FITC fluorophores, respectively. (**A**), Negative control using only the fluorescence labeled secondary antibodies. (**B**), Spermatozoa with rabbit anti-PPP1CC2 and sheep anti-PPP1R2 antibodies. Phase contrast (PH). Scale bar = 20 μm.

**Figure 7:**
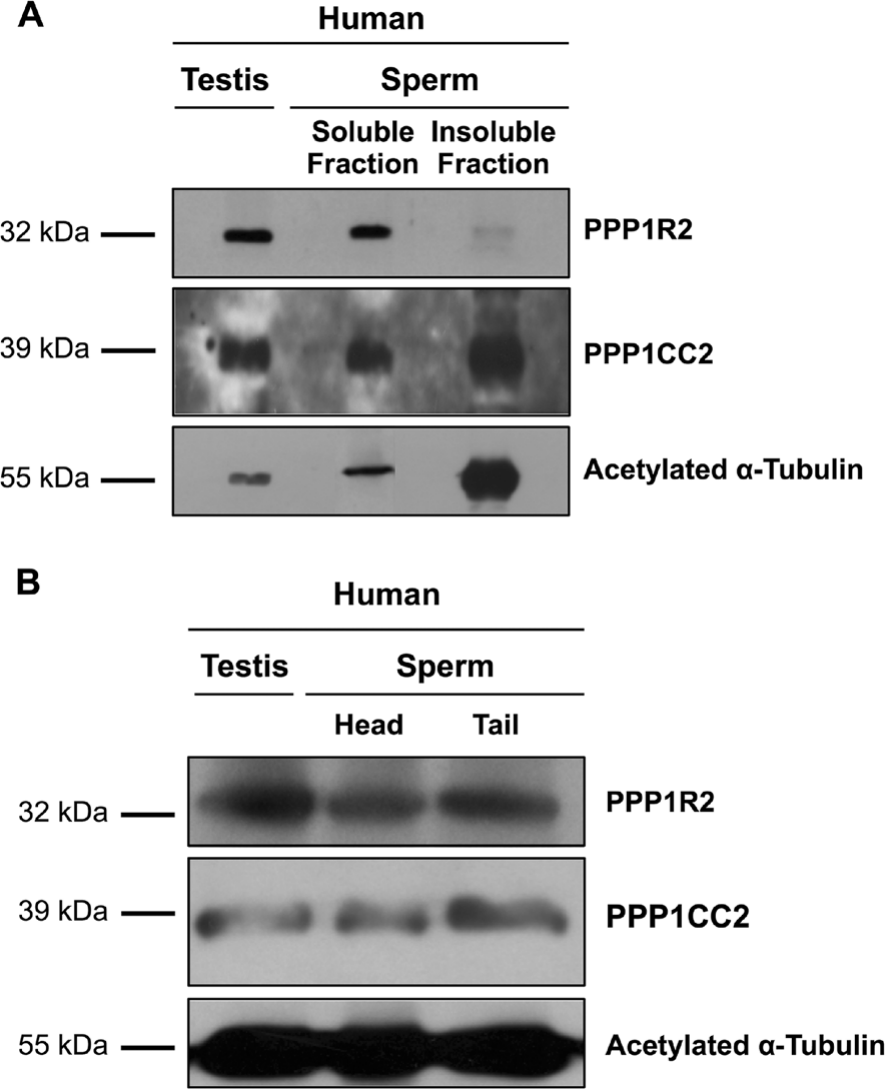
**PPP1R2/PPP1R2P3 are present in human testis and sperm extracts.** (**A**), PPP1R2/PPP1R2P3 and PPP1CC2 presence in testis and in the RIPA supernatant (soluble fraction) and pellet (insoluble fraction) of sperm lysates, was analyzed using sheep anti-PPP1R2 and rabbit anti-PPP1CC2 antibodies. 100 μg of both fractions were loaded in a SDS-PAGE gel. Acetylated α-tubulin was used as a loading control. (**B**), A human sperm sample (2 × 10^8^ sperm cells) was sonicated to disrupt head and tail bond, and subjected to a sucrose gradient. Both pools were verified by phase contrast (PH). 100 μg of both fractions were loaded in a SDS-PAGE gel. After transfer, the blot was probed with sheep anti-PPP1R2 and rabbit anti-PPP1CC2 antibodies. Acetylated α-tubulin was used as a loading control.

Immunocytochemistry was used to study the subcellular localization of PPP1R2 and PPP1R2P3 and their co-localization with PPP1CC2 in mature human spermatozoa. Experiments were performed using sheep anti-PPP1R2 (detects PPP1R2 and PPP1R2P3) and CBC502 antibodies (Figure 6). Results showed that PPP1R2 and PPP1R2P3 are present along the flagellum, in the mid-piece, principal piece, except the end-piece, and also in the head, more specifically in the equatorial and post-acrosomal regions (Figure 6B). PPP1CC2 localization is similar, co-localizing to the same regions as seen with PPP1R2. A negative control using only secondary antibodies showed that the staining was specific (Figure 6A). The PPP1CC2 localization corroborated a previous report and the localization is consistent with a role for PPP1 in sperm forward and hyperactivated motility and possibly in the acrosome reaction [3-5,7,35]. To support the immunolocalization data, RIPA soluble extracts and insoluble fractions were resolved by SDS-PAGE and immunoblotted (Figure 7A). The results showed that PPP1R2 and PPP1R2P3 were mainly present in the soluble extracts. Although RIPA lysis buffer is a stringent buffer we still detected some PPP1R2 protein in the insoluble fraction. In contrast, PPP1CC2 is present in both fractions but more abundantly in the insoluble fraction. These results are consistent with PPP1CC2 being tightly attached to the axoneme.

Furthermore, tail and head preparations were also obtained using a sucrose gradient. In this separation although heads are kept intact, tails are demembranated and only the axoneme structure is maintained, thus all soluble proteins present in the tail are removed. The results clearly demonstrate that PPP1R2 and PPP1R2P3, as well as PPP1CC2, are present in the spermatozoa tail and head and in similar amounts (Figure 7B).

### Phosphorylation of PPP1R2/PPP1R2P3 in human sperm

**Figure 8:**
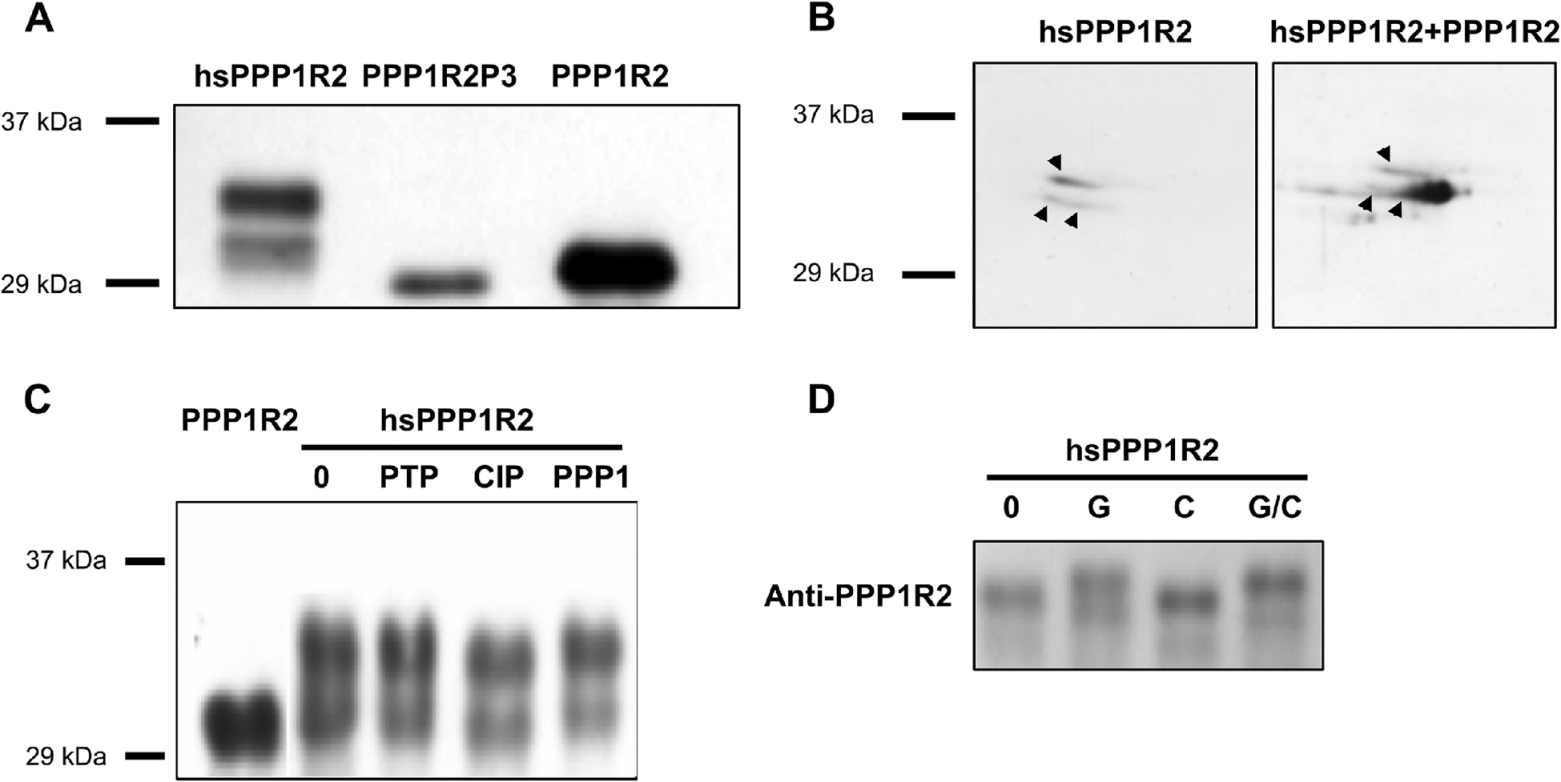
**Western blot analysis of endogenous PPP1R2/PPP1R2P3 from human sperm.** (**A**), Comparison of endogenous human sperm PPP1R2/PPP1R2P3, with recombinant PPP1R2 and PPP1R2P3, using sheep anti-PPP1R2 antibody. hsPPP1R2, heat-stable human sperm extract (both PPP1R2 and PPP1R2P3). Recombinant PPP1R2P3 and PPP1R2 are shown as positive controls. (**B**), Anti-PPP1R2 Western blot of 2D separation of hsPPP1R2 human sperm extract. Left panel, heat-stable human sperm extract; right panel, heat-stable human sperm extract supplemented with recombinant PPP1R2. Arrowheads indicate the three spots with different molecular mass and pI (**C**), hsPPP1R2 was incubated in the presence of different phosphatases, in the respective buffers at 30ºC for 3 hrs. 0, no phosphatase, control; PTP, incubation with Protein Tyrosine Phosphatase 1B; CIP, incubation with Calf Intestinal Phosphatase; PPP1, incubation with PPP1CC1. Recombinant PPP1R2 is shown as a positive control. (**D**), hsPPP1R2 was incubated in the presence of GSK3 (G) or CK2 (C), or both (G/C), as previously described. Resulting changes in protein migration, probably reflecting alterations in phosphorylation, were detected by Western blot analysis using the sheep anti-PPP1R2 antibody.

Western blotting of human sperm heat-treated extracts revealed, depending on the samples, two bands migrating slower than the purified PPP1R2 and PPP1R2P3 (Figure 8A). To investigate the nature of those bands, human sperm heat-treated extracts were resolved by 2D-PAGE, revealing three isolated spots of different molecular mass and pI, suggesting the existence of different phosphorylated forms of PPP1R2 and PPP1R2P3 (Figure 8B, arrowheads). Besides threonine and serine, PPP1R2 can also be tyrosine phosphorylated [36,37]. The human sperm heat-treated extract was dephosphorylated by treatment with protein tyrosine phosphatase 1B (PTP), calf intestinal phosphatase (CIP) and PPP1CC1. Under the conditions used, only CIP, a nonspecific phosphatase, could dephosphorylate the PPP1R2 and PPP1R2P3, with an increase in electrophoretic mobility. This band however did not run at the same molecular mass as recombinant PPP1R2 (Figure 8C). When sperm PPP1R2 was incubated with GSK3 or CK2 or both, only GSK3 was able to induce a mobility shift (Figure 8D).

## Conclusions

The data here presented clearly shows that PPP1R2 is present in human sperm, based on immunoreactivity and mass spectrometry analysis. This supports the model that the PPP1 inhibitor activity identified several years ago can be accounted for by PPP1R2 [3,5]. In the past, *PPP1R2* mRNAs of 1.2 kb, 1.4–1.7 kb, 2.4–2.7 kb and 4 kb have been found in rat and rabbit testis [28]. These mRNAs strictly correspond to the polyadenylation (polyA) signals present in PPP1R2. Further, a new highly expressed testis-specific 0.9–1.1 kb message, presumably spliced from the *PPP1R2* gene was also found in rabbit and rat. This message only was detected after 50 days and not before 20 days of age in rats [28,37]. Another report identified PPP1R2 protein in heat-stable extracts of bull testis and mouse testis and sperm [35]. Of particular relevance we identified, by mass spectrometry, a second PPP1R2-related protein in human sperm, termed PPP1R2P3. This was previously considered to be a pseudogene because it lacks introns, has no parental promoter, and seems to be integrated in the genome at a random new location. Pseudogenes originate from retrotransposition activity, so they have truncated 5’UTR due to the low processivity of the reverse transcriptase, and direct repeats at both ends [38]. Testis is an organ where many pseudogenes are expressed as mRNA and proteins that have been shown to actively participate in spermatogenesis or other germ cell functions [39,40]. Transcription in testis, when compared to other somatic tissues, tends to activate alternative promoters, which are otherwise imperfect or weak [40,41]. The apparent function of pseudogenes in testis germ cells could be a way to facilitate the appearance of new genes from the parental ones [39].

Previous work suggests that PPP1CC2 plays a key role in sperm motility. This enzyme is likely regulated by a mechanism involving reversible phosphorylation of a PPP1R2 mediated by GSK3 kinase [3,5]. Peptides for PPP1CC2 were recovered in immunoprecipitates of PPP1R2/PPP1R2P3, indicating the presence of complexes between the enzyme and the inhibitors in sperm. Since immunoprecipitation was through antibodies against PPP1R2 the presence of other regulatory subunits were not determined. However, the results support the view that at least a proportion of PPP1 in sperm is likely to be regulated by PPP1R2 proteins. Our results also show that PPP1R2P3 is phosphorylated by CK2 *in vitro*, probably at residues 121, 122 or 127, as shown by the MS-MS spectra, even though the Ser87 is absent. Peptides previously assigned to PPP1R2P3 protein may have been derived also from PPP1R2 [42,43]. Acetylation at Ala2 and phosphorylation at Ser121 and Ser122 were observed by high throughput shotgun MS analysis in human Jurkat T cell leukemia and embryonic kidney (HEK293) cell lines [42,43]. These regions (Ala2, Ser121 and Ser122) are similar in both PPP1R2 and PPP1R2P3.

PPP1R2 interaction with PPP1C involves two primary motifs, ^145^KLHY^148^ and ^43^KSQKW^47^. Other points of contact in PPP1R2 are the N-terminal SILK motif (^12^KGILK^16^, in humans) that possibly initiates the binding, and the C-terminal acidic stretch, required for the PPP1 activation by GSK3 [28,44,45]. We show for the first time that PPP1R2P3 has all the PPP1 binding motifs, but lacks the key phosphorylation sites Thr73 and Ser87. These results are consistent with a model whereby PPP1R2P3 forms a complex with PPP1C but the activity of this complex would not be regulated by GSK3. The data show that PPP1R2P3 protein binds directly to PPP1CC and that this inhibitor cannot be phosphorylated by GSK3. The presence of this protein variant, which may be an irreversible inhibitor of PPP1CC provides for a novel mode of regulating the catalytic activity of the phosphatase (Figure 4).

Intrasperm localization analysis showed that both PPP1R2/PPP1R2P3 and PPP1CC2 co-localize in the same sperm subcellular structures. That is, PPP1CC2 is probably bound to the axoneme along with PPP1R2/PPP1R2P3 (Figures 5, 6 and 7). A previous report showed that PPP1 in *Chlamydomonas* is anchored to the central pair apparatus of the axoneme, associated with the C1 microtubule and to a lesser extent to the outer doublet microtubules. This axonemal localization suggests that PPP1 can control both dynein arms and thereby kinetic activity of the flagellum [46]. Moreover, PPP1C has a two-fold higher activity in immotile bovine caput epididymal sperm compared to mature motile caudal sperm which is consistent with it being directly involved in sperm motility [3,5]. The regulation of sperm motility by this phosphatase should involve its inhibitors. We hypothesize that in the initial segments of the epididymis (caput) sperm are immotile due to the reversible activity of the PPP1R2/PPP1CC2 complexes, while in the latter segments of the epididymis (cauda), PPP1R2P3 substitutes for PPP1R2 as an irreversible inhibitor of PPP1CC2 thereby resulting in sperm motility. These independent interactions may be occurring concurrently, since peptides specific for both proteins were identified in ejaculated sperm. Since sperm are terminally differentiated cells, essentially devoid of transcriptional and translational activity, regulation occurs through pre-existing proteins and not through new synthesis of proteins. It is possible that PPP1R2P3 could be bound to another protein that may keep it from binding to PPP1CC2 in immotile caput sperm, in a similar manner to what is suggested to occur with sds22 (PPP1R7) in caput sperm [47]. Also, other PPP1CC2 complexes exist in sperm as dimers or trimers: such as PPP1R11/PPP1R7/PPP1CC2 [6,22].

This mechanism of controlling sperm motility by PPP1CC2 and GSK3 may operate only in mammals, since other vertebrate classes do not have this alternatively spliced phosphatase [33]. Furthermore, PPP1R2P3 protein, which is only present in primates, could lead to a novel regulatory mechanism for regulating PPP1CC2 activity that might have evolved only in this order. Determination of the validity of these possibilities needs further studies. Work is in progress in our laboratory to determine the mechanistic role of PPP1R2 and PPP1R2P3 in regulating PPP1CC2 activity and thereby motility and fertility of spermatozoa.

## Methods

### Yeast two-hybrid

Methods for yeast two-hybrid screening of a human testis cDNA library using human *PPP1CC* have been previously described [15,48,49]. DNA sequence analysis was performed using an ABI PRISM 310 Genetic Analyser (Portugal Applied Biosystems, Porto, Portugal). The DNA sequences obtained were compared to the NCBI database, using the BLAST algorithm (http://BLAST.ncbi.nlm.nih.gov/). The multiple sequence alignments were performed using the ClustalW program from Ensembl.

### PPP1R2 and PPP1R2P3 cloning, expression and purification

The cDNA of *PPP1R2P3* was ligated into the pET28c (Novagen, Madison, Wisconsin, USA) expression vector using *EcoRI* and *XhoI* restriction sites, adding a histidine tag (His-tag) to the N-terminus of the protein. The pET-PPP1R2P3 sequence was verified and the plasmid transformed into *E. coli* strain *Rosetta* (DE3) (Novagen, Madison, Wisconsin, USA). The expression of His-tag PPP1R2P3 was induced with 1 mM isopropyl β-D-1-thiogalactopyranoside (IPTG) for 3 hrs at 37ºC and the protein purified using a Ni-NTA resin (QIAGEN, Dusseldorf, Germany) according to the supplier’s instructions. Briefly, the cells were lysed in 10 mM imidazole, sodium phosphate buffer, pH 8.0, centrifuged at 15000 g for 30 min at 4ºC, and the supernatant was applied to the resin. The resin was washed with 20 mM imidazole and the His-tag PPP1R2P3 was eluted with 500 mM imidazole. The protein was further purified by 12% SDS-PAGE. A portion of the lane containing PPP1R2P3 was stained with Coomassie blue and the region containing the remaining protein was excised, washed three times with water, cut into smaller pieces and 1 ml of 100 mM Tris-HCl, pH 8.5, 0.1% SDS was added before freezing at -20ºC overnight. The slurry was frozen and thawed three times and then passed through a 0.22 μm filter membrane. The gel-free filtrate was then dialyzed against 4 × 500 ml of 10 mM Tris-HCl, pH 7.5 buffer for 24 hrs at 4ºC. The protein concentration of recombinant His-PPP1R2P3 was determined by BCA® assay (Fisher Scientific, Loures, Portugal). *PPP1R2P3* cDNA was also ligated into the pTACTAC expression vector [50] in the *NdeI* and *XbaI* restriction sites, the sequence verified and then transformed into *Rosetta* strain. The expression of PPP1R2P3 was induced with 0.4 mM IPTG for 3 hrs at 37ºC. The protein was partially purified by boiling the bacterial extract (here on referred to as recombinant PPP1R2P3) as previously described for PPP1R2 [25]. *PPP1R2* cDNA was likewise ligated in pTACTAC expression vector [50] and recombinant protein purified in the same way as for PPP1R2P3 (here on referred to as recombinant PPP1R2). *PPP1R2P3* cDNA was excised using *EcoRI* and *XhoI* from pET-PPP1R2P3 and subcloned in pACT-2 vector (Clontech, Saint Germain-en-Laye, France) for co-transformation.

### Yeast co-transformation with plasmid DNA

Yeast competent AH109 cells were co-transformed with pACT-PPP1R2P3 and pAS2-PPP1CA, pAS2-PPP1CC1, pAS2-PPP1CC2 or pAS2-PPP1CC2end, by the lithium acetate method [15]. pAS2-PPP1CC constructs used were previously described [15]. For negative and positive controls pAS2-1/pACT-2 and pVA3-1/pTD1-1 vectors were used, respectively. Afterwards, the transformation mixture was plated on selective media containing X-α-Gal and incubated at 30ºC to check for MEL1 expression as indicated by the appearance of a blue color (Clontech, Saint Germain-en-Laye, France).

### Blot overlay analysis

For blot overlay analysis, 0.3 μg of commercial PPP1R2 (NEB, New England Biolabs, Herts, UK) and recombinant His-PPP1R2P3 were resolved by SDS-PAGE and then transferred to a nitrocellulose membrane. Blots were overlaid with purified PPP1CC1 or PPP1CC2 (25 pmol/mL) diluted in Tris buffered saline with Tween-20/BSA [48,51] and detected with the antibodies CBC3C (against the C-terminal of PPP1CC, which detects both isoforms [52]) or CBC502 (specific for the C-terminal of PPP1CC2), both raised in rabbit. Immunoreactive bands were revealed by incubating with horseradish peroxidase conjugated anti-rabbit secondary antibody and developed by enhanced chemiluminescence (ECL, GE Healthcare, Amersham Biosciences Europe GmbH, Freiburg, Germany).

### Phosphatase activity assays

The IC50 values of PPP1R2 and PPP1R2P3 for purified PPP1CC1 and PPP1CC2 isoforms were determined using [32P]phosphorylase a as substrate. The substrate was prepared from phosphorylase b (Sigma-Aldrich Química, S.A., Sintra, Portugal) using [γ-32P]ATP (3000 Ci/mmol, GE Healthcare, Amersham Biosciences Europe GmbH, Freiburg, Germany) and phosphorylase kinase (Sigma-Aldrich Química, S.A., Sintra, Portugal) as previously described [48]. An appropriate range of concentrations of commercial PPP1R2 and His-PPP1R2P3 were incubated with the purified PPP1C isoforms and the phosphatase activity determined. The IC50 was calculated using the BioDataFit 1.02 software (Chang Bioscience, Castro Valley, California, USA).

### Sperm extracts

Ejaculated sperm were collected from healthy donors by masturbation into an appropriate sterile container. Spermograms were performed by experienced technicians and only samples with normal parameters were used [53]. For all the methods, sperm was washed three times in 1× PBS. For immunoprecipitation, sperm was lysed in 1× RIPA buffer (radioimmunoprecipitation buffer, Millipore Iberica S.A.U., Madrid, Spain) supplemented with protease (10 mM benzamidine, 1.5 μM aprotinin, 5 μM pepstatin A, 2 μM leupeptin, 1 mM PMSF) and phosphatase (1 mM sodium fluoride, 2.5 mM sodium pyrophosphate, 50 mM beta-glycerophosphate, 1 mM sodium orthovanadate) inhibitors, sonicated 3× for 10 sec and centrifuged at 16000 g for 20 min, at 4ºC. The supernatant was collected and heat-stable extracts were prepared by immersing the sample in a boiling water bath for 30 min, chilled on ice for 2 min and centrifuged at 16000 g for 20 min, at 4ºC. The final supernatant was used in the subsequent steps. For Western blot, both the supernatant (soluble fraction) and the pellet (insoluble fraction) were resuspended in 1% SDS [54].

For the preparation of heads and tails, washed sperm were briefly sonicated and the detachment checked by phase contrast microscopy (PH) using an Olympus IX81 epifluorescence microscope, equipped with appropriate software (Olympus Portugal - Opto-Digital Tecnologias, S.A., Lisboa, Portugal). Sperm were directly applied onto a sucrose step gradient (1.8 M, 2.02 M and 2.2 M) to separate heads from tails, by centrifuging at 5000 g for 1 hr and fractions corresponding to tails and heads collected. Subsequently fractions were centrifuged at 16000 g, 10 min, resulting in a pellet free of sucrose and proteins were dissolved in 1% SDS. For immunocytochemistry, washed sperm was used directly on the coverslips. Human testicular biopsy was also prepared using 1% SDS and the same protocol as for sperm samples, but previously homogenized using a tissue homogenizer. Testicular biopsy was collected in Centro Hospitalar de Coimbra, Portugal during a procedure to collect organs for transplantation from a brain death 35-years-old adult man and the biopsy was diagnosed as “normal spermatogenesis” based on histopathological analysis.

### Consent

Written informed consent, for the use of sperm samples, was obtained from the donors for publication and any accompanying images. A copy of the written consent is available for review by the Editor-in-Chief of this journal. The study was conducted in accordance with the guidelines of the “Helsinki Declaration”.

Testicular biopsies for research purposes are covered by the legislation of the Portuguese Constitution (decreto-lei nº274/99 of July 22, 1999: “*It is permitted the dissection of corpses, or parts of them, of national citizens, stateless persons and foreign residents in Portugal, as well as extraction of parts, tissues or organs when the deceased has explicitly declared in life's will that his body can be used for purposes of teaching and scientific research.*”).

### Phosphorylation of PPP1R2 and PPP1R2P3

Recombinant PPP1R2, PPP1R2P3 or human sperm heat extract of native PPP1R2/PPP1R2P3 (about 200 ηg of inhibitor protein in 50 mM Tris-HCl pH 7.5, 0.1 mM EGTA and 0.03% Brij-35) were phosphorylated by GSK3β or CK2 (Calbiochem, MERCK, Darmstadt, Germany) or both kinases as previously described [28]. The phosphorylation reaction was run at 30ºC for 90 min and then terminated with the addition of 4× SDS loading buffer. Two separate 12% gels were run, one with 1/10 of the reaction volume and the other with the remaining 9/10 of the reaction volume. The 1/10 reaction gel was transferred and analyzed by Western blot while the 9/10 reaction gel was dried and autoradiographed.

### Immunoprecipitation

RIPA supernatant sperm extracts were pre-cleared using dynabeads protein G (Life Technologies S.A., Madrid, Spain). A direct immunoprecipitation approach was performed with 1 μg of sheep anti-PPP1R2 or rabbit anti-PPP1R2 pre-incubated with Dynabeads® Protein G during 1 hr at 4ºC with rotation. After incubation, pre-cleared sperm extracts were applied to the antibody-dynabeads complex and incubated overnight with rotation at 4ºC. After washing three times with 1× PBS in 3% BSA for 10 min with rotation at 4ºC, beads were resuspended in loading buffer and boiled.

### Mass spectrometry

For mass spectrometry analysis, immunoprecipitates were resolved by 10% SDS-PAGE along with purified positive controls. Gels were stained with Coomassie blue colloidal (Sigma-Aldrich Química, S.A., Sintra, Portugal). In brief, gels were fixed with a fixation solution (40% methanol and 10% acetic acid) during 1 hr, washed with distilled water and then transferred to the Coomassie blue colloidal staining solution for 1 hr. After staining, the gels were washed with distilled water and afterwards destained with 25% methanol until bands were visualized.

Bands were excised directly from the gel using a spatula and completely destained. In-gel digestion was performed overnight at 37°C with trypsin (Promega, Madison, Wisconsin, USA) in 10 mM HCl and 50 mM ammonium hydrogen carbonate (NH_4_HCO_3_) at pH 7.8. Resulting peptides were extracted once with 100 μl of 1% formic acid (FA), and twice with 100 μl of 5% FA, 50% acetonitrile (ACN). Extracts were combined and ACN was removed *in vacuo*. For LC-MS analysis, a final volume of 40 μl was prepared by addition of 1% FA. Electrospray tandem mass spectrometry (ESI-MS/MS) was performed on an Orbitrap Velos instrument (Thermo Scientific, Bremen, Germany). Fragment ions were generated by low-energy collision-induced dissociation (CID) on isolated ions with a fragmentation amplitude of 0.5 V. MS spectra were summed from four individual scans ranging from m/z 300–1500 with a scanning speed of 8.100 (m/z)/s. MS/MS spectra were a sum of two scans ranging from m/z 100–2800 at a scan rate of 26.000 (m/z)/s. Generated data were imported to ProteinScape™, a proteomics data platform (Bruker Daltonik GmbH, Bremen, Germany) and analyzed using MASCOT (version 2.2.0*,* Matrix Science*,* London, UK) search algorithm with search parameters as follows: precursor ion tolerance of 1.2 and 0.3 Da for MS/MS spectra. Proteins were considered to be identified if the Mascot score (ProteinScape™) was higher than 65.

### Western blotting

Extracts were mass normalized using BCA® assay (Fisher Scientific, Loures, Portugal). Immunoprecipitates and extracts were resolved by 10% SDS-PAGE. Proteins were subsequently electrotransferred onto nitrocellulose membranes and immunodetected with the appropriate antibodies, using ECL detection (GE Healthcare Spain, Madrid, Spain). The primary antibodies used in this study included sheep polyclonal anti-PPP1R2 (1:100), the rabbit CBC502 (1:2000, against the C-terminal of PPP1CC2), the rabbit CBC3C (1:1000, against the C-terminal and detects both PPP1CC isoforms) and the loading controls, mouse monoclonal anti-β-tubulin (1:500, Life Technologies S.A., Madrid, Spain) and mouse monoclonal acetylated-α-tubulin (1:2000, Life Technologies S.A., Madrid, Spain). The secondary antibodies used were horseradish peroxidase-conjugated anti-rabbit (1:5000), anti-sheep (1:1000) and anti-mouse (1:5000) IgGs for ECL detection (GE Healthcare, Amersham Biosciences Europe GmbH, Freiburg, Germany).

### Immunocytochemistry

An aliquot of washed diluted sperm (25 μl) was placed onto a glass coverslip pre-coated with 100 μg/ml poly-L-ornithine, and dried at room temperature, in a six-well plate containing one coverslip per well. To each well 1 ml of 4% paraformaldehyde in 1× PBS was gently added and left to stand for 10 min. Subsequently, sperm sample was washed twice with 1 ml 1× PBS for 10 min. For permeabilization, 1 ml of 1:1 methanol/acetone solution was added for 2 min and then the samples washed twice with 1 ml 1× PBS for 10 min and blocked for 1 hr with 3% BSA in 1× PBS, before incubation with primary antibodies (rabbit CBC502, 1:250 and sheep anti-PPP1R2, 1:100) for 2 hrs at room temperature. After three washes with 1× PBS, the fluorescently labeled secondary antibodies anti-rabbit Texas-Red, 1:300 (MolecularProbes, Eugene, USA) and anti-sheep FITC, 1:50 (DAKO, Glostrup, Denmark) were added and the coverslips incubated for 2 hrs. Finally, three washes with 1× PBS were performed and coverslips were mounted on microscope glass slides with one drop of anti-fading reagent containing DAPI for nucleic acid staining (Vectashield, Vector Laboratories Burlingame, California, USA). Images were acquired using an Olympus IX81 epifluorescence microscope and digital camera, equipped with the appropriate software (Olympus Portugal-Opto-Digital Tecnologias, S.A., Lisboa, Portugal).

### 2D-PAGE analysis

The human sperm heat-stable extracts (hsPPP1R2) or hsPPP1R2 plus 10 ηg of recombinant PPP1R2 were acetone precipitated and the pellets were resuspended in 250 μl of 2D rehydration solution (8 M Urea/ 2 M Thiourea/ 2% CHAPS/ 0.002% of bromophenol blue) and supplemented with 2.5 μl of IPG buffer (in the 4–7 pH range) and 14 mg of DTT. The samples were pipetted into a strip holder and the electrophoresis was started (1 hr at 30 V, 2 hrs at 150 V, 1 hr at 500 V, 1 hr at 1000 V and 2 hrs at 8000 V). After the second dimension on 12% gels, samples were analyzed by Western blot.

### Dephosphorylation of human sperm PPP1R2/PPP1R2P3

The hsPPP1R2 extracts were incubated overnight with either protein tyrosine phosphatase 1B (Upstate, Millipore Iberica S.A.U., Madrid, Spain) at 37ºC, calf intestinal phosphatase (NEB, New England Biolabs (UK), Herts, UK) at 37ºC or PPP1CC1, at 30ºC, with the respective assay buffers. The reactions were stopped with the addition of 4× SDS loading buffer and analyzed by Western blot.

## Abbreviations

GSK3: Glycogen synthase kinase 3; CK2: Casein kinase 2; PPP1: Phosphoprotein phosphatase 1; PPP1C: Catalytic subunit of PPP1; PPP1R2: PPP1 regulator 2; PPP1R2P3: PPP1R2 pseudogene 3; IPTG: Isopropyl β-D-1-thiogalactopyranoside; ECL: Enhanced chemiluminescence; RIPA: Radioimmunoprecipitation buffer; PH: Phase contrast microscopy; FA: Formic acid; ACN: Acetonitrile; ESI-MS/MS: Electrospray mass spectrometry; CID: Low-energy collision-induced dissociation; FITC: Fluorescein isothiocyanate; DAPI: 4',6-diamidino-2-phenylindole; DTT: Dithiothreitol; IPG: Immobilized pH gradient; EST: Expressed sequence tag; MGC: Mammalian gene collection; HEK293: Human embryonic kidney 293 cells; pI: Isoelectric point; aa: Amino acid; IP: Immunoprecipitation.

## Competing interests

The authors declare that they have no competing interests.

## Authors' contributions

Performed the sequence alignment: LKG. Conceived and designed the experiments: LKG, MFe, APV, WW, MF and EFCS. Performed the experiments: LKG, MFe, APV, WW and TM. Analyzed the data: LKG, MFe, APV, WW and TM. Contributed with reagents/materials/analysis tools: KM, SV, DLB, OABCS, MF and EFCS. Wrote the first draft: LKG. Edited the first draft: MFe, SV, DLB, OABCS and MF. All authors read and approved the final manuscript.
